# Nitrogen-containing bisphosphonates inhibit cell cycle progression in human melanoma cells

**DOI:** 10.1038/sj.bjc.6602052

**Published:** 2004-07-27

**Authors:** A-M Forsea, C Müller, C Riebeling, C E Orfanos, C C Geilen

**Affiliations:** 1 Ist Clinic of Dermatology, Colentina Hospital, ‘Carol Davila’ University of Medicine and Pharmacy, Bucharest, Romania; 2 Department of Dermatology, Charité University Medical Center Berlin, Campus Benjamin Franklin, Fabeckstrasse 60-62, Berlin-Dahlem D-14195, Germany; 3 Department of Biological Chemistry, Weizmann Institute of Science, Rehovot 76100, Israel

**Keywords:** melanoma, bisphosphonates, apoptosis, cell cycle

## Abstract

Cutaneous melanoma is one of the highly malignant human tumours, due to its tendency to generate early metastases and its resistance to classical chemotherapy. We recently demonstrated that pamidronate, a nitrogen-containing bisphosphonate, has an antiproliferative and proapoptotic effect on different melanoma cell lines. In the present study, we compared the *in vitro* effects of three different bisphosphonates on human melanoma cell lines and we demonstrated that the two nitrogen-containing bisphosphonates pamidronate and zoledronate inhibited the proliferation of melanoma cells and induced apoptosis in a dose- and time-dependent manner. Moreover, cell cycle progression was altered, the two compounds causing accumulation of the cells in the S phase of the cycle. In contrast, the nonaminobisphosphonate clodronate had no effect on melanoma cells. These findings suggest a direct antitumoural effect of bisphosphonates on melanoma cells *in vitro* and further support the hypothesis of different intracellular mechanisms of action for nitrogen-containing and nonaminobisphosphonates. Our data indicate that nitrogen-containing bisphosphonates may be a useful novel therapeutic class for treatment and/or prevention of melanoma metastases.

Melanoma is a highly malignant tumour and its propensity to metastasise together to its resistance to therapy in later stages make it the most aggressive skin cancer (Serrone and Hersey, 1999; Soengas and Lowe, 2003). The high mortality rate of malignant melanoma, the poor efficacy of chemotherapy in advanced stages of the disease and the high toxicity of the classical regimens have stimulated intensive research for new alternatives for the therapy of melanoma. Various studies suggest that an intrinsic resistance to apoptosis can be one important mechanism by which melanoma cells escape therapeutic control (Soengas and Lowe, 2003). Therefore, new therapeutical strategies that bypass this resistance are necessary.

Bisphosphonates are a class of synthetic analogues of the endogenous pyrophosphate, which are well established in the treatment of osteoclast-mediated bone diseases such as osteoporosis, Paget's disease of the bone and tumour-induced osteolysis (Fleisch, 1997; Finley, 2002). They have been used in medical practice for more than three decades for their antidemineralising effects. Recently, an increasing body of evidence from both *in vitro* and *in vivo* studies suggests that bisphosphonates may also have a specific antitumoural action (Clezardin, 2002; Padalecki and Guise, 2002; Green, 2003). Thus, bisphosphonates have been shown to inhibit proliferation, induce cell cycle changes and/or induce apoptosis in various types of human tumour cells, especially in those with preferential spread to bone, such as multiple myeloma, breast or prostate carcinoma cells (Shipman *et al*, 1997; Senaratne *et al*, 2000; Hiraga *et al*, 2001; Lee *et al*, 2001; Sonnemann *et al*, 2001; Iguchi *et al*, 2003; Oades *et al*, 2003).

For the nitrogen-containing bisphosphonates, this antiproliferative and proapoptotic effect appears to be related to their ability to inhibit the enzymes of the mevalonate pathway, especially farnesyl pyrophosphate (FPP) synthase (Luckman *et al*, 1998; Benford *et al*, 1999; Senaratne *et al*, 2002). Consequently, bisphosphonates prevent the synthesis of higher isoprenoids such as geranylgeranyl pyrophosphate (GGPP) and FPP, which are necessary for the post-translational processing (prenylation) of different signalling molecules, including monomeric G proteins of the Ras and Rho families. For these families of small GTPases, the prenyl residues act as membrane anchors essential for their activation and further interaction with other signalling molecules (Bar-Sagi and Hall, 2000; Aznar and Lacal, 2001). Ras and Rho protein families are key regulators of a variety of cellular processes, ranging from reorganisation of the cytoskeleton to transcriptional regulation and control of cell growth and survival (Aznar and Lacal, 2001). When their expression and activation escape the control mechanisms, small GTPases play an essential part in promoting tumorigenesis and tumour metastasing (Fritz *et al*, 1999; Clark *et al*, 2000; Pruitt and Der, 2001). Therefore, their inactivation by inhibition of prenylation could explain at least in part the antitumoral effects described for bisphosphonates. On the contrary, bisphosphonates that lack a nitrogen atom, such as clodronate, appear to have no effect on the mevalonate pathway, but rather reduce cell viability by metabolism to inactive analogues of ATP, and consequently, by disruption of the ATP-dependent processes of the cell (Rogers *et al*, 1996; Rogers *et al*, 1999).

We have previously demonstrated that the nitrogen-containing bisphosphonate pamidronate is able to induce apoptosis and to inhibit proliferation in melanoma cells *in vitro* (Riebeling *et al*, 2002). Melanoma metastasises less often to bone, but it is an aggressive tumour with high metastatic potential and marked resistance to the currently available antitumour therapy strategies. New therapy alternatives are urgently required, and we addressed the question of the possible benefit of bisphosphonates in the adjuvant therapy of melanoma. Besides the well-known pamidronate, a wide range of newer bisphosphonates with higher antiresorptive effect have been introduced in practice (Green, 2001; Fleisch, 2002; Widler *et al*, 2002). However, the relationship between antiresorptive potency, mechanism of action and cellular effects of bisphosphonates has not been completely elucidated. Moreover, to what extent bisphosphonates of different pharmacological classes differ in their effects in tumour cells or if higher antiresorptive potency implies a stronger effect against tumour cell growth is still a matter of debate.

The present study aims to compare the effect of three different bisphosphonates, with different postulated mechanisms of action and different antiresorptive potencies, on cell proliferation, cell cycle progression and cell survival in melanoma *in vitro*. We have chosen for this purpose the nonaminobisphosphonate clodronate, widely used in the treatment of cancer-induced osteolytic disease, and two nitrogen-containing bisphosphonates, pamidronate and the newly developed zoledronate, the most potent antiresorptive agent known to date.

## MATERIAL AND METHODS

### Reagents

Pamidronate (Aredia®) (3-amino-1-hydroxy-propyldiene-1,1-bisphosphonate) and zoledronate (Zometa®) (2-(imidazole)-1-yl) hydroxyethylidene-1,1-bisphophonate) were obtained from Novartis Pharma (Nürnberg, Germany). Clodronate (dichlorohydromethylene-diphosphonic acid) was purchased from Sigma (Munich, Germany).

All three bisphosphonates were dissolved in distilled water and filter sterilised (sterile filters, B Braun, Melsungen, Germany). Stock solutions (at final concentrations of 21.5 mM for pamidronate, and 100 mM for zoledronate and clodronate) were aliquoted and kept at −20°C for long-term storage.

Caspase-3 inhibitor was purchased from Alexis (Grünberg, Deutschland). Cells were pretreated with the inhibitor 1 h prior to stimulation.

Dulbecco's modified Eagle's medium (DMEM) was purchased from Invitrogen (Karlsruhe, Germany). Further cell culture reagents were obtained from Seromed-Biochrom (Berlin, Germany). All other reagents were obtained from Sigma (Munich, Germany) unless stated otherwise.

### Cell culture

The melanoma cell line A375 (CRL-1619), derived from primary tumour, was purchased from American Type Culture Collection (Manassas, VA, USA). The melanoma cell population M186 was obtained by surgical intervention from a patient with histologically confirmed melanoma metastases. Melanoma cells were grown in 75 cm^2^ culture flasks (Nunc, Wiesbaden, Germany) in DMEM supplemented with 10% heat-inactivated foetal calf serum, 100 U ml^−1^ penicillin and 100 *μ*g ml^−1^ streptomycin, in a 5% CO_2_ atmosphere at 37°C.

### Proliferation assay

Proliferation was assessed using the crystal violet staining method (Wieder *et al*, 1998). Subconfluent melanoma (60 000 cells well^−1^) were treated in 24-well plates with the indicated agents or corresponding solvents as control. After the indicated incubation time, culture medium was removed, cells were rinsed with phosphate-buffered saline (PBS) to wash off nonadherent cells and the remaining cells were fixed with 0.1 M glutaraldehyde in PBS for 30 min at room temperature. Subsequently, cells were washed with PBS and then stained by incubation with 0.2 mM crystal violet in PBS for 30 min at room temperature. Unbound dye was washed away in deionised water for 15 min and 0.2% Triton X-100 was added to release the bound dye. After 1 h of incubation, 100 *μ*l supernatant of each sample was transferred to 96-well microtitre plate and the extinction at 570 nm was measured using an ELISA photometer. Extinction values of vehicle-treated control cells were set at 100% and the rate of proliferation of bisphosphonate-treated cells was calculated as the percent of controls.

### Cytotoxicity assay

Cytotoxicity was determined using the Cytotoxicity Detection Kit (LDH) Roche Diagnostics, (Mannheim, Germany). After incubation of 80 000 cells well^−1^ in 24-well plates, for up to 24 h plates were centrifuged at 300 **g** for 5 min. A measure of 50 *μ*l of the resulting supernatant were transferred into a microtitre plate and lactate dehydrogenase (LDH) activity was determined by the addition of substrate solution. Formation of the formazan salt was measured at 490 nm using an ELISA photometer. Extinction values of control cells were set at 100% and the rate of LDH release from the treated cells was calculated as the percent of controls.

### Cell death detection

Induction of apoptosis was measured using the ‘Cell death detection ELISA^PLUS^’ kit from Roche Diagnostics (Mannheim, Germany), which detects oligonucleosomes released into the cytoplasm of cells during apoptosis, by means of a combination of anti-histone and anti-DNA antibodies, as described (Wieder *et al*, 1998).

Cells were seeded at 80 000 cells well^−1^ in 24-well plates and left to adhere overnight. Subsequently, cells were treated as indicated, after which the plates were centrifuged at 300 **g** for 5 min. The supernatant was cautiously removed and the cells further incubated with lysis buffer for 30 min at room temperature. After centrifugation at 300 **g** for 10 min, 20 *μ*l from the resulting supernatants were transferred to a streptavidin-coated microtitre plate, supplemented with 80 *μ*l of immunoreagent solution (containing biotin-coupled anti-histone antibodies and peroxidase-coupled anti-DNA antibodies) and incubated for 2 h at room temperature under moderate shaking.

After incubation, the wells were rinsed with incubation buffer, supplied with 100 *μ*l substrate solution per well and further incubated for 10 min at room temperature, under protection from light. The extinction of the samples at 405 nm was measured using an ELISA photometer. Extinction values of control samples were set at 100% and DNA fragmentation of treated cells was calculated as the percent of control.

### Measurement of caspase-3/7 activity

Capase-3/7 activity was measured by proteolytic cleavage of the fluorogenic substrate Z-DEVD-R110 using the Apo-One™ Homogeneous Caspase-3/7 Assay (Promega, Madison, WI, USA). Cells were treated for 24 h in 96-well plates with the corresponding bisphosphonates or vehicle as control, at concentrations as indicated. Apo-One™ Homogeneous Caspase-3/7 buffer containing Z-DEVD-R110 diluted 1 : 100 was added to the cells and incubated at room temperature. The activity was measured fluorimetrically with an excitation wavelength of 499 nm and an emission wavelength of 521 nm after 90 min. Caspase-3/7 activity was determined and expressed as the percentage of control.

### Cell cycle analysis

The distribution of cells in different phases of the cell cycle after treatment with bisphosphonates was analysed by measuring the DNA content of cells using FACS analysis after nuclear staining with propidium iodide. Melanoma cells were seeded at 80 000 cells well^−1^ in six-well plates. After 48 h, they were treated as indicated and subsequently washed with PBS, trypsinised and harvested in culture medium. All washes and cell solutions were pooled and centrifuged at 200 **g** for 5 min. The cell pellet was resuspended in PBS and 1 × 10^6^ cells of each sample were collected, washed in ice-cold PBS and fixed in ice-cold 70% ethanol in PBS (v v^−1^) at −20°C over night.

For the analysis of DNA content, samples were thawed and centrifuged at 400 **g** for 5 min. The pellet was washed once with 1 ml PBS, and then incubated with 1 ml of 2% propidium iodide and 20% RNase A in PBS, for at least 30 min at room temperature, protected from light. After incubation, the cell suspension was analysed for red fluorescence with a FACSCalibur flowcytometer (Becton Dickinson, Heidelberg, Germany). DNA histograms were created using Cell Quest™ software, version 3.0 for Apple Macintosh (Becton Dickinson), where 20 000 events sample^−1^ were analysed. The relative distribution of cells in the phases of the cell cycle was calculated with ModFitLT software, version 2.0 for Apple Macintosh (Becton Dickinson).

### Statistical analysis

Statistical significance was determined using the Student's *t*-test, with SigmaStat 2.03 software. *P*<0.05 was considered significant.

## RESULTS

### Nitrogen-containing bisphosphonates inhibit melanoma cell proliferation

**Figure 1:**
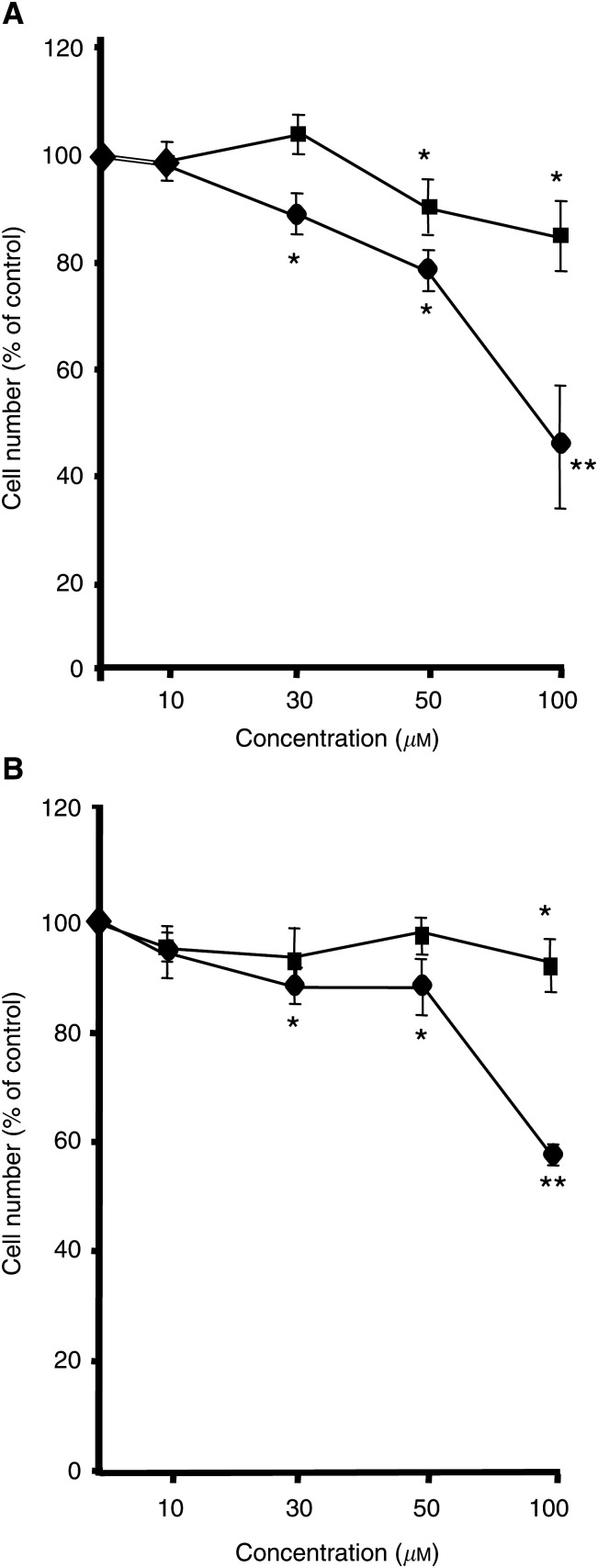
Effects of bisphosphonates on melanoma cell proliferation. A375 (**A**) and M186 (**B**) melanoma cells were incubated for 24 h with the indicated doses of pamidronate (squares) or zoledronate (circles). Cell proliferation was evaluated using the crystal violet technique. Four independent experiments were performed in quadruplicate with similar results. One representative experiment is shown. Results are given as % of controls±s.d. (*n*=4) (^*^P<0.05; ^**^P<0.01).

In order to investigate the effect of bisphosphonates on melanoma cell growth, melanoma cell lines A375 and M186 were treated with increasing concentrations of pamidronate, zoledronate and clodronate for 24 h. The number of cells was determined using the crystal violet method. In both cell lines, pamidronate as well as zoledronate treatment resulted in a dose-dependent decrease in cell number (Figure 1

). In A375 cells, (Figure 1A) a significant reduction in cell number was observed after treatment with 50 *μ*M pamidronate, and reached the maximum after treatment with at 100 *μ*M pamidronate (84% of control). Higher concentrations of pamidronate were not able to induce a further decrease in cell number. Zoledronate was more effective in inhibiting cell growth. The cell number was significantly reduced to 89% of control at a concentration of 30 *μ*M and further decreased to 45% of control at 100 *μ*M zoledronate. A similar effect of bisphosphonate treatment was observed in M186 cells (Figure 1B). A slight, yet still significant reduction of cell number was observed for pamidronate at a concentration of 100 *μ*M (88% of control), and a stronger effect was observed for zoledronate, beginning at a concentration of 30 *μ*M and reaching a maximum at 100 *μ*M with 57% of control.

In contrast, incubation of both A375 and M186 cells with the nonaminobisphosphonate clodronate, at concentrations ranging from 100 to 1000 *μ*M, failed to induce a significant decrease in cell number within 24 h (data not shown).

### Nitrogen-containing bisphosphonates induce apoptosis in melanoma cell lines

DNA fragmentation as a marker of apoptosis was evaluated by means of an ELISA technique in A375 and M186 cells after 24 h of incubation with increasing concentrations of pamidronate, zoledronate or clodronate.

**Figure 2:**
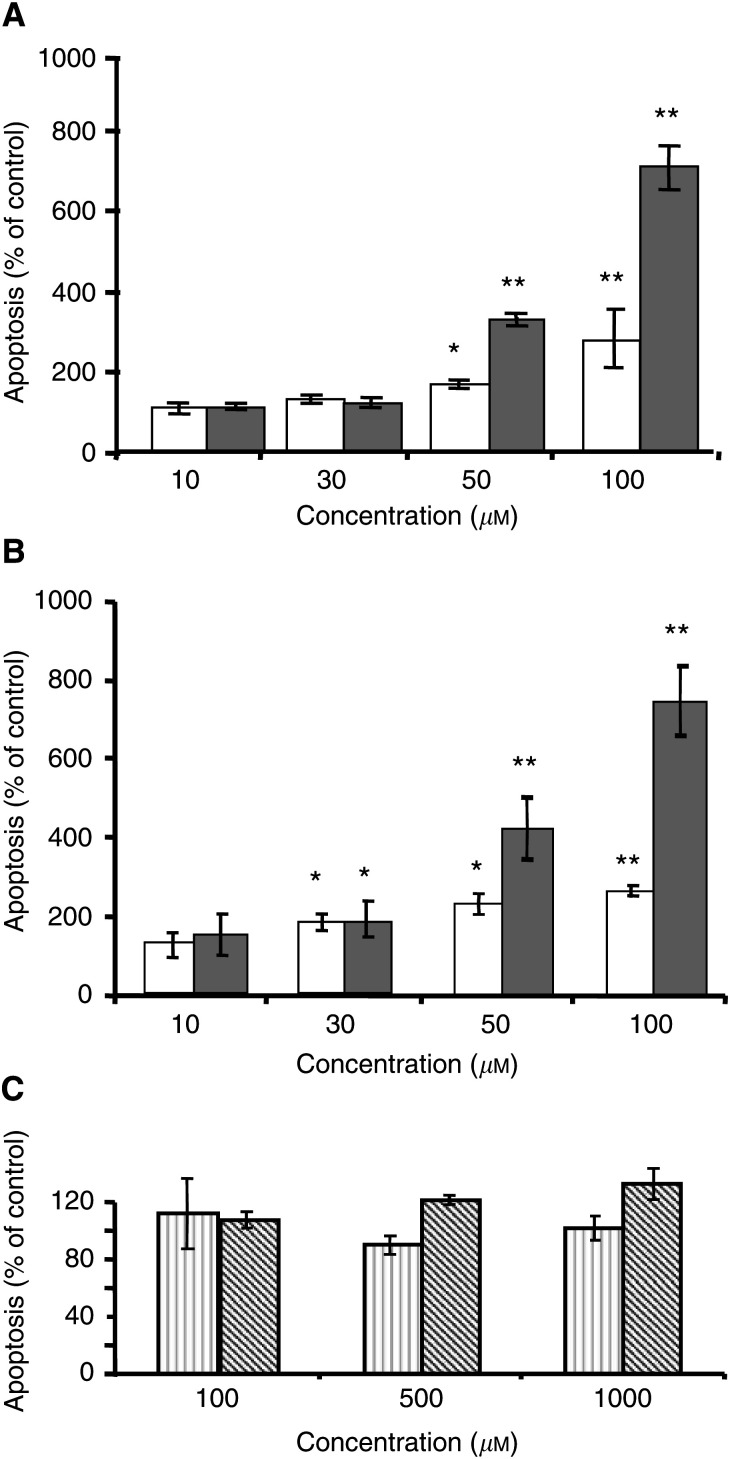
Effect of bisphosphonates on the induction of apoptosis in melanoma cells. Preconfluent A375 (**A**) and M186 (**B**) melanoma cells were treated with the indicated concentrations of zoledronate (white columns), or pamidronate (grey columns) for 24 h. (**C**) A375 (light grey columns) and M186 (hatched columns) cells were treated for 24 h with clodronate in the indicated concentrations. DNA fragmentation was measured using the ‘Cell death detection ELISA^PLUS^’ as described under Materials and methods. Four independent experiments were performed in quadruplicate, with similar results. One representative experiment is shown. Results are given as % of controls±s.d. (*n*=4) (^*^P<0.05; ^**^P<0.01).

A dose-dependent induction of DNA fragmentation was observed after both zoledronate and pamidronate treatment in the two-cell populations that were studied. In A375 cells, a significant effect was detectable at concentrations of 50 *μ*M and was further increased at 100 *μ*M (Figure 2A

). In M186 cells, increased DNA fragmentation was first detected at a concentration of 30 *μ*M (Figure 2B). In both cell lines, 100 *μ*M pamidronate had a stronger effect in inducing apoptosis, with DNA fragmentation reaching 711% of control in A375 cells, and 746% of control in M186 cells, while, using 100 *μ*M zoledronate, DNA fragmentation reached only 280% of controls in A375 cells and 247% in M186 cells. In contrast, A375 and M186 cells treated for 24 h with clodronate in concentrations ranging from 100 to 1000 *μ*M showed no significant effect on DNA fragmentation (Figure 2C).

**Figure 3:**
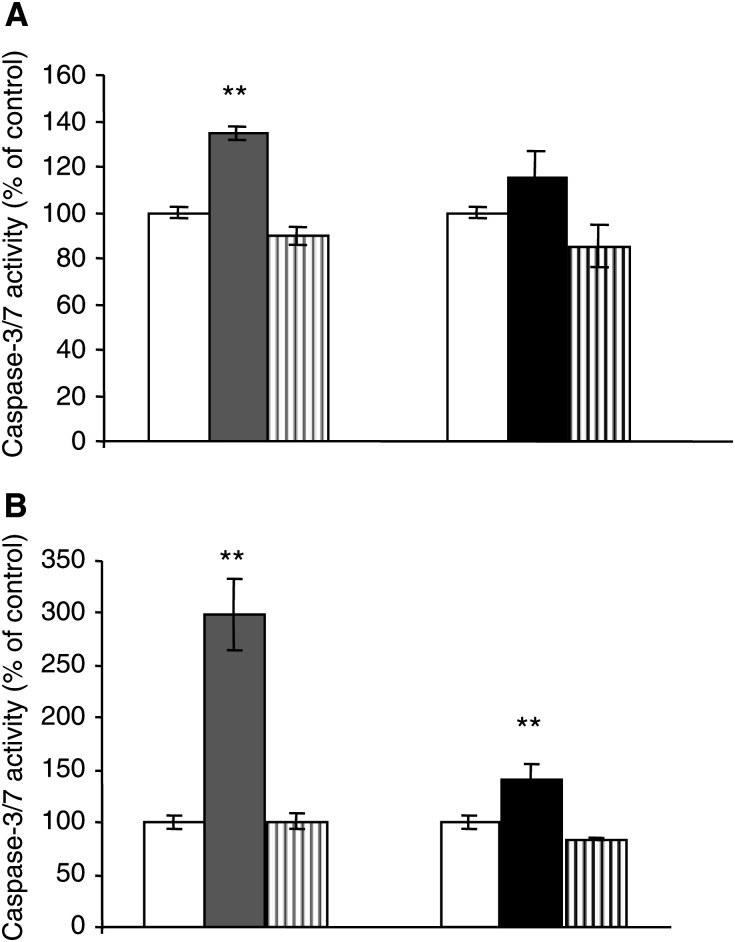
Nitrogen-containing bisphosphonates induce caspase-3/7 activity in melanoma cells. Preconfluent A 375 (**A**) and M186 (**B**) cells were treated with vehicle control (white bars), 100 *μ*M pamidronate (grey columns) or 100 *μ*M zoledronate (black columns) for 24 h. For specific activation of caspase-3, cells were pretreated with the respective caspase-3 inhibitor 1 h prior to stimulation and then treated for 24 h with 100 *μ*M pamidronate or zoledronate in combination with the inhibitor (square bars). Caspase-3/7 activity was determined with the Apo-One™ homogeneous caspase-3/7 assay as described under Materials and methods. Three independent experiments were performed in quadruplicate, with similar results. One representative experiment is shown. Results are given as % of control±s.d. (*n*=4) (^*^P<0.05; ^**^P<0.01).

The activity of the execution caspase-3 or -7 is a further marker of apoptosis. The data obtained for DNA fragmentation correlated well with the caspase activity measured in bisphosphonate-treated melanoma cells. A375 and M186 cells were treated for 24 h with 100 *μ*M pamidronate or zoledronate, respectively, after which the activity of caspase-3/7 was measured. The data obtained on caspase activation further support a stronger proapoptotic effect of pamidronate, in comparison to zoledronate. (Figure 3

). Treatment of the cells with clodronate showed no effect on caspase-3/7 activity (data not shown).

A possible unspecific cytotoxic effect of bisphosphonates on melanoma cells was investigated by measuring the extracellular release of LDH, following bisphosphonate treatment. No increase of LDH release in comparison with controls was found in M186 and A375 cells treated for 24 h with zoledronate or pamidronate in a concentration range between 10 and 100 *μ*M, while treatment with the nonaminobisphosphonate clodronate, in concentrations ranging from 100 to 1000 *μ*M, induced a moderate but significant increase in extracellular LDH activity measured after 24 h (data not shown). Thus, nitrogen-containing bisphosphonates are able to induce apoptosis in a dose-dependent manner in melanoma cells, while the nonaminobisphosphonate clodronate appears to induce necrosis without apoptotic effect.

**Figure 4:**
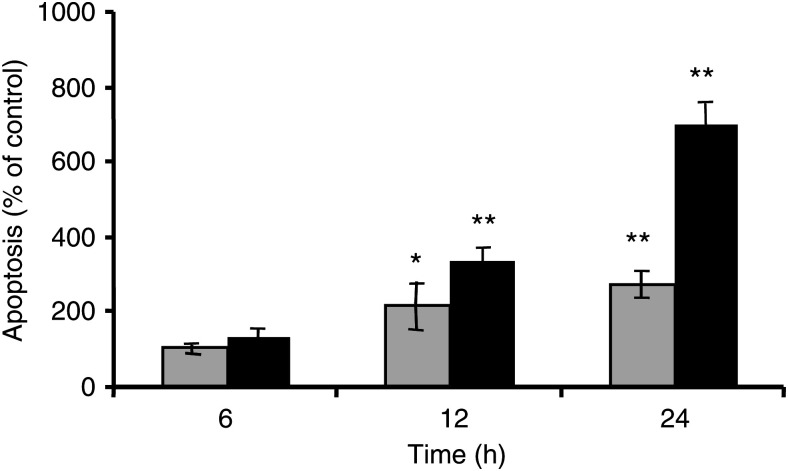
Time dependency of bisphosphonate-induced apoptosis in melanoma cells. Preconfluent A375 melanoma cells were treated with the indicated concentrations of zoledronate (grey columns) or pamidronate (black columns), or vehicle for 6, 12 and 24 h. At each time point, DNA fragmentation was measured using the ‘Cell death detection ELISA^PLUS^’ as described under Materials and methods. Three independent experiments were performed in quadruplicate, with similar results. One representative experiment is shown. Results are given as % of controls±s.d. (*n*=4) (^*^P<0.05; ^**^P<0.01).

Further on, we investigated the relationship between the apoptotic effect of bisphosphonates and the duration of the treatment. A375 melanoma cells were incubated for 6, 12 and 24 h with 100 *μ*M pamidronate or zoledronate. A significant increase in DNA fragmentation was observed after 12 h of incubation with both bisphosphonates (Figure 4

), and increased further markedly after 24 h of treatment, reaching up to 700% of controls for pamidronate and 315% for zoledronate. Thus, apoptosis induced by bisphosphonates is dependent on both concentration and duration of treatment.

### Bisphosphonates inhibit progression of melanoma cells through the cell cycle

In order to study the effect of bisphosphonates on cell cycle progression of melanoma cells, FACS analysis of the DNA content was used to investigate the distribution of A375 and M186 cells in the phases of the cell cycle. Cells were treated for 24 h with increasing concentrations of pamidronate, zoledronate or clodronate.

**Figure 5:**
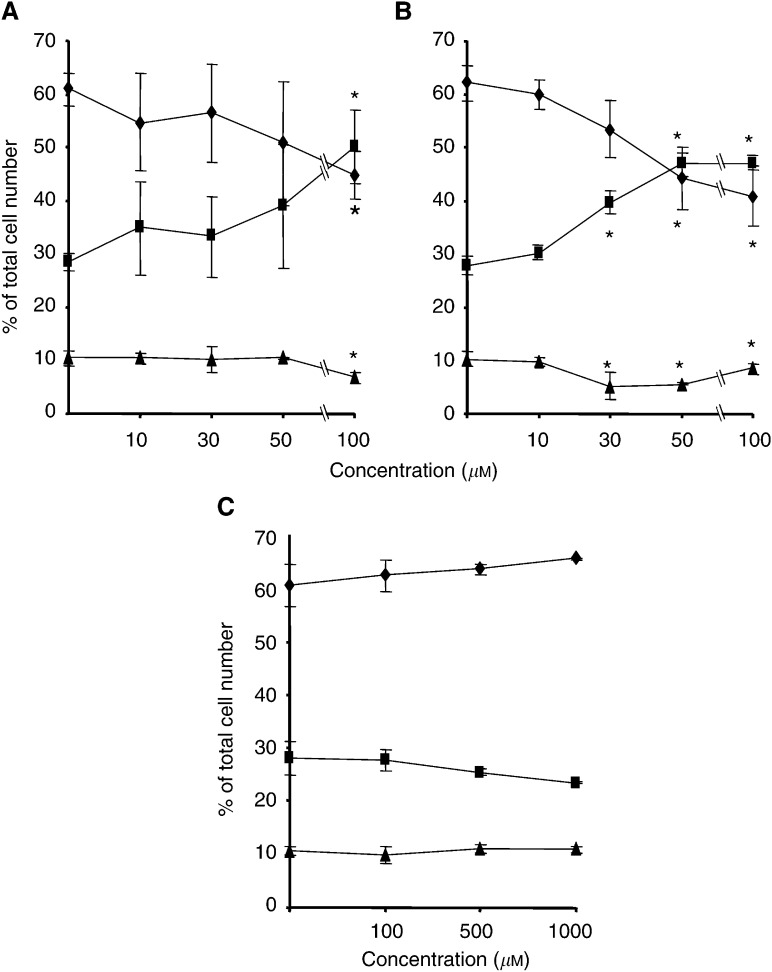
Distribution in various phases of the cell cycle of cultured melanoma cells after bisphosphonate treatment. A375 cells were treated with (**A**) pamidronate, (**B**) zoledronate or (**C**) clodronate at the indicated concentrations for 24 h. DNA content of cells was determined by propidium iodide staining using FACS analysis, and the distribution of cells in G0/G1 (diamonds), S (squares) and G2/M (triangles) phases of the cell cycle is shown. Results are given as the percentage of cells in G0/G1, S and G2/M phases as determined by ModFitLT software (version 2.0). Values represent the mean of three independent experiments±s.d. (*n*=3) (^*^P<0.05).

Vehicle-treated cultures exhibited a distribution of cells in the phases of the cell cycle typical for proliferating cells, with an average of 61% of cells having a 2*n* DNA content, corresponding to G0/G1 phase, 10% of cells having a 4*n* DNA content (G2/M) and 28% showing a DNA content between 2*n* and 4*n*, corresponding to the S phase (Figure 5

).

Cells treated with pamidronate or zoledronate in a concentration range of 10–100 *μ*M showed comparable dose-dependent alterations in cell cycle distribution, with an increase in number of cells in S phase accompanied by a reduction in the proportion of cells in G0/G1 and G2/M phases (Figure 5A and B). Zoledronate was more potent in inducing changes in cell cycle distribution, its effects starting at concentrations of 30 *μ*M, while pamidronate significantly altered the distribution of cells in the cell cycle phases only at the highest concentration. The maximum effect was seen for both drugs at a concentration of 100 *μ*M, with an increase in the proportion of cells in S phase from 28 to 49% for pamidronate and to 47% for zoledronate.

In contrast, treatment of cells with clodronate at concentrations 10 times higher showed a different pattern, with the tendency of an increase in the proportion of cells in G0/G1 phase (Figure 5C).

## DISCUSSION

At present, bisphosphonates are emerging as new potential antitumoral drugs. While most of the studies on bisphosphonates concentrate on tumours with preferential spreading to bone, such as breast or prostate cancer, we were the first to show that pamidronate can induce apoptosis in melanoma cells (Riebeling *et al*, 2002). In order to further investigate the potential benefit of bisphosphonates in the treatment of melanoma, the present study compares the effect of these compounds on proliferation, cell cycle progression and apoptosis induction in melanoma cell lines. Three bisphosphonates, with different structure, antiresorptive potency and postulated mechanism of action, namely pamidronate, zoledronate and clodronate, were analysed.

Our results indicate that both nitrogen-containing bisphosphonates pamidronate and zoledronate are able to decrease cell proliferation *in vitro* in a dose-dependent manner. Inhibition of cell growth was not the result of necrosis, as no significant release of LDH from the cells was measured after treatment with the two bisphosphonates. At the same time, proliferation inhibition cannot be explained only by induction of apoptosis, since the antiproliferative capacity did not correlate to the proapoptotic effect of these nitrogen-containing bisphosphonates. Both pamidronate and zoledronate induced DNA fragmentation in the two studied melanoma cells lines, A375 and M186, in a dose- and time-dependent manner, but pamidronate had a stronger proapoptotic effect. Consistently, pamidronate induced a stronger activation of the execution caspase-3/7. This caspases activation supported the specificity of the proapoptotic effect of bisphosphonates.

In contrast, the nonaminobisphosphonate clodronate, even at concentrations 10 times higher, had no significant effect on cell number and induction of apoptosis in cultured melanoma cells. However, in higher dose, clodronate caused a slight increase in LDH activity, suggesting some cytolytic effect.

These differences observed in the activity of the three bisphosphonates may reflect the difference in the mechanism of action between the nitrogen-containing and nonaminobisphosphonates. The stronger antiproliferative and/or proapoptotic effect of nitrogen-containing bisphosphonates compared to nonaminobisphosphonates was also reported in other cell types such as macrophages (Benford *et al*, 1999; Rogers *et al*, 1999), breast cancer cells (Senaratne *et al*, 2000), multiple myeloma (Shipman *et al*, 1998; Shipman *et al*, 2000) or colon adenocarcinoma (Suri *et al*, 2001), and this effect appears to be related to the ability of nitrogen-containing bisphosphonates to inhibit the mevalonate pathway and thereby the prenylation of signalling proteins such as the small GTPases. Both pamidronate and zoledronate were shown to inhibit specifically the enzyme FPP synthase (Bergstrom *et al*, 2000; Dunford *et al*, 2001), and the depletion of cellular pools of GGPP and FPP has been demonstrated to be a key mechanism in the induction of apoptosis and reduction of cell viability by nitrogen-containing bisphosphonates (Benford *et al*, 1999; Jagdev *et al*, 2001; Reszka *et al*, 2001). Consistently, in melanoma, we previously demonstrated that the apoptotic effect of pamidronate could be reversed by supplementation of cells with GGPP and FPP precursors, which circumvents the bisphosphonate-induced inhibition of isoprenoid synthesis (Riebeling *et al*, 2002). However, the exact mechanisms by which inactivation of small GTPases leads to the induction of apoptosis have not been elucidated to date. In melanoma, the apoptotic action of nitrogen-containing bisphosphonates involves caspase-3 activation and as shown previously (Riebeling *et al*, 2002) is not influenced by bcl-2 overexpression.

In contrast to nitrogen-containing bisphosphonates, clodronate does not inhibit isoprenoid synthesis (Reszka *et al*, 1999). Rather, it has been reported that nonaminobisphosphonates can induce cell death by metabolism to toxic nonhydrolysable analogues of ATP (Rogers *et al*, 1996) and consequently by disrupting the energy-requiring processes of the cells. In the present study, the lack of effect of clodronate on cell survival *in vitro* (even at high concentrations) may suggest that this mechanism has no functional significance in melanoma cell lines.

Although the two nitrogen-containing bisphosphonates differed in their capacity to induce apoptosis and to inhibit cell proliferation, a prominent effect of both was the alteration of the progression of melanoma cells through the phases of the cell cycle. Measurement of the cellular DNA content by FACS analysis revealed that both zoledronate and pamidronate caused accumulation of cells in the S phase of the cycle in the two melanoma cell lines studied, with a corresponding decrease in the number of cells in G1 and G2/M phases (Figure 5). This effect was dose dependent and stronger using zoledronate, which induced significant alterations of the cell cycle progression starting at the concentration of 30 *μ*M; in comparison, pamidronate had a significant effect on cell cycle progression only at the concentration of 100 *μ*M. The mechanism of these changes in the cell cycle is not clear. A similar delay in the S-phase progression has been documented in myeloma (Aparicio *et al*, 1998; Iguchi *et al*, 2003), prostate cancer cells (Lee *et al*, 2001) or keratinocytes (Reszka *et al*, 2001) treated with nitrogen-containing bisphosphonates, and this could be related to the inhibition of prenylation of small GTPases. Both Ras and Rho proteins are known as important regulators of the cell cycle (Hirai *et al*, 1997; Olson *et al*, 1998; Pruitt and Der, 2001; Welsh *et al*, 2001), and in consequence their inactivation via inhibition of prenylation by bisphosphonates could explain the alterations of the cell cycle observed after treatment with these compounds. It would also be consistent with the observation that other inhibitors of the mevalonate pathway, such as statins, also induce comparable cell cycle changes (Vogt *et al*, 1997; Naderi *et al*, 1999).Very recently it was shown, for myeloma cells, that S-phase cell cycle arrest induced by nitrogen-containing bisphosphonates is linked to mitogen-activated protein kinase (MAPK) cascade activation (Iguchi *et al*, 2003).

Consistent with the lack of effect of clodronate on cell proliferation and apoptosis, this compound also failed to significantly alter cell cycle progression, even at the higher concentrations.

Zoledronate is considered the most effective antidemineralising agent available on the market, being about 100 times more potent then pamidronate in inhibiting bone resorption *in vivo* (Widler *et al*, 2002). However, in our study pamidronate was more efficient than zoledronate in inducing apoptosis in both melanoma cell lines studied. In contrast, zoledronate proved to be more potent in altering the cell cycle progression of cells and in inhibiting cell proliferation.

These results suggest that zoledronate affects mostly cell growth, while pamidronate acts rather by inducing cell death. Similar differences in the actions of the two agents have also been reported in some of the studies in breast (Boissier *et al*, 2000)or prostate cancer cells (Lee *et al*, 2001). Pamidronate has also been shown to be more effective in inducing cell death than other bisphosphonates with higher antiresorptive potency (Coxon *et al*, 2000; Senaratne *et al*, 2000; Benford *et al*, 2001; Suri *et al*, 2001). The lack of correlation between the antiresorptive potency of bisphosphonates *in vivo* and their antitumoral effect *in vitro* appears to be dependent on both compound and cell type, and may be explained, for example, by selective inhibition by bisphosphonates of additional enzymes of the mevalonate pathway (van Beek *et al*, 1999; Rogers *et al*, 2000; Thompson *et al*, 2002) or possible additional mechanisms of action of bisphosphonates, for example, MAPK signalling (Iguchi *et al*, 2003).

One objection point mentioned in most of the studies on bisphosphonates is the doses at which the antitumoral effect is achieved. In our study, inhibition of proliferation, cell cycle progression changes and induction of apoptosis in cultured melanoma cells by the two aminobisphosphonates were observed at concentrations ranging from 10 to 100 *μ*M. Similar concentrations of aminobisphosphonates have been reported to act antiproliferative and/or proapoptotic in other types of tumour cells, for example, myeloma (Shipman *et al*, 1997), breast cancer (Senaratne *et al*, 2000)or prostate cancer (Lee *et al*, 2001). It is however not clear if these high concentrations may also be achieved *in vivo*, at least using the current dosage and treatment regimens. As bisphosphonates are rapidly removed from circulation following administration and accumulate in the bone, primary tumour cells or visceral metastases would be most likely exposed to only much lower doses of bisphosphonates, probably in the range 1–5 *μ*M (Daley-Yates *et al*, 1991; Berenson *et al*, 1997). Different treatment regimens or new bisphosphonates analogues with less affinity for the bone may be necessary in future to solve this problem.

In summary, we demonstrate that nitrogen-containing bisphosphonates are effective antitumour agents in melanoma *in vitro*, as they inhibit proliferation, cause an S-phase delay in the cell cycle progression and induce apoptosis in melanoma cells. These encouraging results need to be confirmed by *in vivo* studies and further investigation is required to clarify the exact mechanism of the antineoplasic action of bisphosphonates, as well as their most effective structure and dosing regimen, in order to establish the possible benefit of these compounds in the adjuvant treatment of melanoma.

## References

[bib1] Aparicio A, Gardner A, Tu Y, Savage A, Berenson J, Lichtenstein A (1998) *In vitro* cytoreductive effects on multiple myeloma cells induced by bisphosphonates. Leukemia 12: 220–229 9519785 10.1038/sj.leu.2400892

[bib2] Aznar S, Lacal JC (2001) Rho signals to cell growth and apoptosis. Cancer Lett 165: 1–10 11248412 10.1016/s0304-3835(01)00412-8

[bib3] Bar-Sagi D, Hall A (2000) Ras and Rho GTPases: a family reunion. Cell 103: 227–238 11057896 10.1016/s0092-8674(00)00115-x

[bib4] Benford HL, Frith JC, Auriola S, Monkkonen J, Rogers MJ (1999) Farnesol and geranylgeraniol prevent activation of caspases by aminobisphosphonates: biochemical evidence for two distinct pharmacological classes of bisphosphonate drugs. Mol Pharmacol 56: 131–140 10385693 10.1124/mol.56.1.131

[bib5] Benford HL, McGowan NW, Helfrich MH, Nuttall ME, Rogers MJ (2001) Visualization of bisphosphonate-induced caspase-3 activity in apoptotic osteoclasts *in vitro*. Bone 28: 465–473 11344045 10.1016/s8756-3282(01)00412-4

[bib6] Berenson JR, Rosen L, Vescio R, Lau HS, Woo M, Sioufi A, Kowalski MO, Knight RD, Seaman JJ (1997) Pharmacokinetics of pamidronate disodium in patients with cancer with normal or impaired renal function. J Clin Pharmacol 37: 285–290 9115053 10.1002/j.1552-4604.1997.tb04304.x

[bib7] Bergstrom JD, Bostedor RG, Masarachia PJ, Reszka AA, Rodan G (2000) Alendronate is a specific, nanomolar inhibitor of farnesyl diphosphate synthase. Arch Biochem Biophys 373: 231–241 10620343 10.1006/abbi.1999.1502

[bib8] Boissier S, Ferreras M, Peyruchaud O, Magnetto S, Ebetino FH, Colombel M, Delmas P, Delaisse JM, Clezardin P (2000) Bisphosphonates inhibit breast and prostate carcinoma cell invasion, an early event in the formation of bone metastases. Cancer Res 60: 2949–2954 10850442

[bib9] Clark EA, Golub TR, Lander ES, Hynes RO (2000) Genomic analysis of metastasis reveals an essential role for RhoC. Nature 406: 532–535 10952316 10.1038/35020106

[bib10] Clezardin P (2002) The antitumor potential of bisphosphonates. Semin Oncol 29: 33–42 10.1053/sonc.2002.37420 12584693

[bib11] Coxon FP, Helfrich MH, Van't Hof R, Sebti S, Ralston SH, Hamilton A, Rogers MJ (2000) Protein geranylgeranylation is required for osteoclast formation, function, and survival: inhibition by bisphosphonates and GGTI-298. J Bone Miner Res 15: 1467–1476 10934645 10.1359/jbmr.2000.15.8.1467

[bib12] Daley-Yates PT, Dodwell DJ, Pongchaidecha M, Coleman RE, Howell A (1991) The clearance and bioavailability of pamidronate in patients with breast cancer and bone metastases. Calcif Tissue Int 49: 433–435 1818770 10.1007/BF02555856

[bib13] Dunford JE, Thompson K, Coxon FP, Luckman SP, Hahn FM, Poulter CD, Ebetino FH, Rogers MJ (2001) Structure–activity relationships for inhibition of farnesyl diphosphate synthase *in vitro* and inhibition of bone resorption *in vivo* by nitrogen-containing bisphosphonates. J Pharmacol Exp Ther 296: 235–242 11160603

[bib14] Finley RS (2002) Bisphosphonates in the treatment of bone metastases. Semin Oncol 29: 132–138 11894024 10.1053/sonc.2002.31534

[bib15] Fleisch H (2002) Development of bisphosphonates. Breast Cancer Res 4: 30–34 11879557 10.1186/bcr414 PMC138713

[bib16] Fleisch HA (1997) Bisphosphonates: preclinical aspects and use in osteoporosis. Ann Med 29: 55–62 9073324 10.3109/07853899708998743

[bib17] Fritz G, Just I, Kaina B (1999) Rho GTPases are over-expressed in human tumors. Int J Cancer 81: 682–687 10328216 10.1002/(sici)1097-0215(19990531)81:5<682::aid-ijc2>3.0.co;2-b

[bib18] Green JR (2001) Chemical and biological prerequisites for novel bisphosphonate molecules: results of comparative preclinical studies. Semin Oncol 28: 4–10 10.1016/s0093-7754(01)90259-3 11346859

[bib19] Green JR (2003) Antitumor effects of bisphosphonates. Cancer 97: 840–847 12548584 10.1002/cncr.11128

[bib20] Hiraga T, Williams PJ, Mundy GR, Yoneda T (2001) The bisphosphonate ibandronate promotes apoptosis in MDA-MB-231 human breast cancer cells in bone metastases. Cancer Res 61: 4418–4424 11389070

[bib21] Hirai A, Nakamura S, Noguchi Y, Yasuda T, Kitagawa M, Tatsuno I, Oeda T, Tahara K, Terano T, Narumiya S, Kohn LD, Saito Y (1997) Geranylgeranylated rho small GTPase(s) are essential for the degradation of p27Kip1 and facilitate the progression from G1 to S phase in growth-stimulated rat FRTL-5 cells. J Biol Chem 272: 13–16 8995216

[bib22] Iguchi T, Miyakawa Y, Yamamoto K, Kizaki M, Ikeda Y (2003) Nitrogen-containing bisphosphonates induce S-phase cell cycle arrest and apoptosis of myeloma cells by activating MAPK pathway and inhibiting mevalonate pathway. Cell Signal 15: 719–727 12742232 10.1016/s0898-6568(03)00007-x

[bib23] Jagdev SP, Coleman RE, Shipman CM, Rostami HA, Croucher PI (2001) The bisphosphonate, zoledronic acid, induces apoptosis of breast cancer cells: evidence for synergy with paclitaxel. Br J Cancer 84: 1126–1134 11308265 10.1054/bjoc.2001.1727 PMC2363858

[bib24] Lee MV, Fong EM, Singer FR, Guenette RS (2001) Bisphosphonate treatment inhibits the growth of prostate cancer cells. Cancer Res 61: 2602–2608 11289137

[bib25] Luckman SP, Hughes DE, Coxon FP, Graham R, Russell G, Rogers MJ (1998) Nitrogen-containing bisphosphonates inhibit the mevalonate pathway and prevent post-translational prenylation of GTP-binding proteins, including Ras. J Bone Miner Res 13: 581–589 9556058 10.1359/jbmr.1998.13.4.581

[bib26] Naderi S, Blomhoff R, Myklebust J, Smeland EB, Erikstein B, Norum KR, Blomhoff HK (1999) Lovastatin inhibits G1/S transition of normal human B-lymphocytes independent of apoptosis. Exp Cell Res 252: 144–153 10502407 10.1006/excr.1999.4608

[bib27] Oades GM, Senaratne SG, Clarke IA, Kirby RS, Colston KW (2003) Nitrogen containing bisphosphonates induce apoptosis and inhibit the mevalonate pathway, impairing Ras membrane localization in prostate cancer cells. J Urol 170: 246–252 12796698 10.1097/01.ju.0000070685.34760.5f

[bib28] Olson MF, Paterson HF, Marshall CJ (1998) Signals from Ras and Rho GTPases interact to regulate expression of p21Waf1/Cip1. Nature 394: 295–299 9685162 10.1038/28425

[bib29] Padalecki SS, Guise TA (2002) Actions of bisphosphonates in animal models of breast cancer. Breast Cancer Res 4: 35–41 11879558 10.1186/bcr415 PMC138714

[bib30] Pruitt K, Der CJ (2001) Ras and Rho regulation of the cell cycle and oncogenesis. Cancer Lett 171: 1–10 11485822 10.1016/s0304-3835(01)00528-6

[bib31] Reszka AA, Halasy-Nagy J, Rodan GA (2001) Nitrogen-bisphosphonates block retinoblastoma phosphorylation and cell growth by inhibiting the cholesterol biosynthetic pathway in a keratinocyte model for esophageal irritation. Mol Pharmacol 59: 193–202 11160853 10.1124/mol.59.2.193

[bib32] Reszka AA, Halasy-Nagy JM, Masarachia PJ, Rodan GA (1999) Bisphosphonates act directly on the osteoclast to induce caspase cleavage of mst1 kinase during apoptosis. A link between inhibition of the mevalonate pathway and regulation of an apoptosis-promoting kinase. J Biol Chem 274: 34967–34973 10574973 10.1074/jbc.274.49.34967

[bib33] Riebeling C, Forsea AM, Raisova M, Orfanos CE, Geilen CC (2002) The bisphosphonate pamidronate induces apoptosis in human melanoma cells *in vitro*. Br J Cancer 87: 366–371 12177810 10.1038/sj.bjc.6600476 PMC2364216

[bib34] Rogers MJ, Brown RJ, Hodkin V, Blackburn GM, Russell RG, Watts DJ (1996) Bisphosphonates are incorporated into adenine nucleotides by human aminoacyl-tRNA synthetase enzymes. Biochem Biophys Res Commun 224: 863–869 8713136 10.1006/bbrc.1996.1113

[bib35] Rogers MJ, Frith JC, Luckman SP, Coxon FP, Benford HL, Monkkonen J, Auriola S, Chilton KM, Russell RG (1999) Molecular mechanisms of action of bisphosphonates. Bone 24: 73S–79S 10321934 10.1016/s8756-3282(99)00070-8

[bib36] Rogers MJ, Gordon S, Benford HL, Coxon FP, Luckman SP, Monkkonen J, Frith JC (2000) Cellular and molecular mechanisms of action of bisphosphonates. Cancer 88: 2961–2978 10898340 10.1002/1097-0142(20000615)88:12+<2961::aid-cncr12>3.3.co;2-c

[bib37] Senaratne SG, Mansi JL, Colston KW (2002) The bisphosphonate zoledronic acid impairs Ras membrane [correction of impairs membrane] localisation and induces cytochrome *c* release in breast cancer cells. Br J Cancer 86: 1479–1486 11986784 10.1038/sj.bjc.6600297 PMC2375368

[bib38] Senaratne SG, Pirianov G, Mansi JL, Arnett TR, Colston KW (2000) Bisphosphonates induce apoptosis in human breast cancer cell lines. Br J Cancer 82: 1459–1468 10780527 10.1054/bjoc.1999.1131 PMC2363380

[bib39] Serrone L, Hersey P (1999) The chemoresistance of human malignant melanoma: an update. Melanoma Res 9: 51–58 10338334 10.1097/00008390-199902000-00007

[bib40] Shipman CM, Croucher PI, Russell RG, Helfrich MH, Rogers MJ (1998) The bisphosphonate incadronate (YM175) causes apoptosis of human myeloma cells *in vitro* by inhibiting the mevalonate pathway. Cancer Res 58: 5294–5297 9850051

[bib41] Shipman CM, Rogers MJ, Apperley JF, Russell RG, Croucher PI (1997) Bisphosphonates induce apoptosis in human myeloma cell lines: a novel anti-tumour activity. Br J Haematol 98: 665–672 9332325 10.1046/j.1365-2141.1997.2713086.x

[bib42] Shipman CM, Rogers MJ, Vanderkerken K, Van Camp B, Graham R, Russell G, Croucher PI (2000) Bisphosphonates – mechanisms of action in multiple myeloma. Acta Oncol 39: 829–835 11145441 10.1080/028418600750063587

[bib43] Soengas MS, Lowe SW (2003) Apoptosis and melanoma chemoresistance. Oncogene 22: 3138–3151 12789290 10.1038/sj.onc.1206454

[bib44] Sonnemann J, Eckervogt V, Truckenbrod B, Boos J, Winkelmann W, van Valen F (2001) The bisphosphonate pamidronate is a potent inhibitor of human osteosarcoma cell growth *in vitro*. Anticancer Drugs 12: 459–465 11395574 10.1097/00001813-200106000-00007

[bib45] Suri S, Monkkonen J, Taskinen M, Pesonen J, Blank MA, Phipps RJ, Rogers MJ (2001) Nitrogen-containing bisphosphonates induce apoptosis of Caco-2 cells *in vitro* by inhibiting the mevalonate pathway: a model of bisphosphonate-induced gastrointestinal toxicity. Bone 29: 336–343 11595616 10.1016/s8756-3282(01)00589-0

[bib46] Thompson K, Dunford JE, Ebetino FH, Rogers MJ (2002) Identification of a bisphosphonate that inhibits isopentenyl diphosphate isomerase and farnesyl diphosphate synthase. Biochem Biophys Res Commun 290: 869–873 11785983 10.1006/bbrc.2001.6289

[bib47] van Beek E, Pieterman E, Cohen L, Lowik C, Papapoulos S (1999) Nitrogen-containing bisphosphonates inhibit isopentenyl pyrophosphate isomerase/farnesyl pyrophosphate synthase activity with relative potencies corresponding to their antiresorptive potencies *in vitro* and *in vivo*. Biochem Biophys Res Commun 255: 491–494 10049736 10.1006/bbrc.1999.0224

[bib48] Vogt A, Sun J, Qian Y, Hamilton AD, Sebti SM (1997) The geranylgeranyltransferase-I inhibitor GGTI-298 arrests human tumor cells in G0/G1 and induces p21(WAF1/CIP1/SDI1) in a p53-independent manner. J Biol Chem 272: 27224–27229 9341167 10.1074/jbc.272.43.27224

[bib49] Welsh CF, Roovers K, Villanueva J, Liu Y, Schwartz MA, Assoian RK (2001) Timing of cyclin D1 expression within G1 phase is controlled by Rho. Nat Cell Biol 3: 950–957 11715015 10.1038/ncb1101-950

[bib50] Widler L, Jaeggi KA, Glatt M, Muller K, Bachmann R, Bisping M, Born AR, Cortesi R, Guiglia G, Jeker H, Klein R, Ramseier U, Schmid J, Schreiber G, Seltenmeyer Y, Green JR (2002) Highly potent geminal bisphosphonates. From pamidronate disodium (Aredia) to zoledronic acid (Zometa). J Med Chem 45: 3721–3738 12166945 10.1021/jm020819i

[bib51] Wieder T, Orfanos CE, Geilen CC (1998) Induction of ceramide-mediated apoptosis by the anticancer phospholipid analog, hexadecylphosphocholine. J Biol Chem 273: 11025–11031 9556584 10.1074/jbc.273.18.11025

